# Analysis of Flesh Content, Muscle Nutritional Components and Quality of Crayfish Farmed in Paddy Fields in Major Breeding Provinces of China

**DOI:** 10.3390/foods15132277

**Published:** 2026-06-25

**Authors:** Yuanyuan Zhang, Shuaijie Sun, Yue Li, Xueqing Han, Chuang Liu, Wenjin Zhu

**Affiliations:** Henan Academy of Fishery Sciences, Zhengzhou 450044, China; zhangyuan86123@163.com (Y.Z.); 15738816518@163.com (S.S.); liyue19413@163.com (Y.L.); xqhan94@163.com (X.H.); akademoslc@gmail.com (C.L.)

**Keywords:** rice paddy farming, crayfish, flesh content, nutritional components, quality

## Abstract

Rice–crayfish co-culture is a dominant aquaculture mode in China, while the regional heterogeneity in crayfish nutritional quality remains insufficiently clarified. This study collected rice-field-cultured crayfish samples from six major producing provinces (Henan, Hubei, Hunan, Jiangxi, Jiangsu, and Anhui) to systematically evaluate their meat yield, conventional nutritional components, amino acid and fatty acid profiles, and mineral element contents. Significant regional differences were observed in all measured indicators. Jiangsu crayfish showed the highest meat yield (22.97% in females, 20.72% in males), crude protein (18.5%), and total amino acids (16.59 g/100 g). Jiangxi samples had the highest crude fat (0.7%), ash (1.5%), and calcium (1280 mg/kg). Anhui crayfish exhibited the highest polyunsaturated fatty acid (PUFA) content (47.30%). Hubei recorded the highest total eicosapentaenoic acid (EPA) + docosahexaenoic acid (DHA) (22.41%). Henan crayfish were highest in EPA (19.6%) and manganese (3.72 mg/kg), while Hunan samples had the highest iron (9.06 mg/kg). Valine was identified as the first limiting amino acid in all provinces, with secondary limiting amino acids varying regionally. Despite regional differences in specific nutritional indices, crayfish from all six provinces consistently exhibited high protein, low fat, balanced amino acid profiles, abundant unsaturated fatty acids, and rich essential minerals, with all measured nutritional mineral contents within national food safety limits. The findings provided empirical data and a scientific basis for crayfish quality evaluation, origin characteristic analysis, and targeted nutritional optimization of farming formulas, as well as for the differentiated development of regional crayfish industries.

## 1. Introduction

As a novel ecological circular agriculture model in China, the integrated rice–fish farming system has undergone rapid expansion across in major rice-growing regions in recent years. This co-culture system features efficient water resource utilization, multi-yield production per unit field, reduced agrochemical inputs, and improved ecological sustainability, rendering it a vital aquaculture development model across multiple provinces. By the end of 2024, the total area of integrated rice–fish farming in China had reached approximately 3.07 million hectares, yielding over 4.4 million tons of aquatic products. Provinces including Hubei, Anhui, Hunan, Jiangsu, Jiangxi, and Henan each possess a co-culture area of exceeding 66,667 hectares, placing them among the top ten in China [1,2].

Native to the southern United States, the crayfish (*Procambarus clarkii*) was introduced to China from Japan in 1929. With high protein content, low fat and cholesterol levels, and an abundance of various unsaturated fatty acids, it is a nutritious and palatable freshwater product highly favored by consumers [3]. Existing studies have demonstrated that factors such as geographical region, farming model, and feed can affect the growth performance, muscle quality, immune function, physiological condition, and economic returns of aquatic animals [4,5]. Furthermore, the high genetic diversity of pond-cultured crayfish across different regions lead to substantial variations in muscle quality and nutritional value among crayfish populations [6]. However, previous studies often focused on a single factor, and the impact of the interaction between multiple factors such as geographical environment, farming mode, and germplasm on the nutritional quality of crayfish remains unclear. This study focuses on the rice–crayfish co-culture model, assuming that differences in water quality, soil, feeding methods, and feed quality in different regions may lead to changes in the nutritional components of the edible parts of crayfish. The crayfish from paddy fields in six major farming provinces—Jiangsu, Anhui, Hubei, Hunan, Jiangxi, and Henan—were selected as research subjects. Systematic measurements were conducted on their flesh content, conventional nutrients, amino acids, fatty acids, and mineral element concentrations. The study aims to elucidate regional differences in the nutritional quality of rice-field-cultured crayfish and to provide a scientific basis for the evaluation of crayfish germplasm resources, processing and utilization, and the optimization of aquaculture techniques.

## 2. Materials and Methods

From August to September 2024, a total of 300 live crayfish samples per region were collected from rice paddy fields in six provinces of China: Henan, Hubei, Hunan, Jiangxi, Jiangsu, and Anhui. Only crayfish with intact bodies, free from disease and injury, were selected for the determination of meat content, muscle nutrient composition, and mineral content. For each region, 240 healthy, intact crayfish were selected and divided into three biological replicates of 80 crayfish each.

### 2.1. Determination of Meat Yield

The dirt on the body surface of the crayfish was washed off, and the surface was blotted dry with filter paper. The body mass was then weighed and recorded. The head and claws were removed, and the appendages were cut off. The tail was twisted to extract the shrimp thread, and a cut was made along the abdomen with scissors to remove the shell. The abdominal muscle was carefully peeled off using tweezers, and its surface was blotted dry before weighing. The meat yield was calculated using the following formula:

Meat yield (%) = Abdominal muscle mass (g)/Body weight (g) × 100%

### 2.2. Determination of Proximate Composition

The proximate composition include moisture, ash, crude fat, and crude protein. The moisture content was determined using the atmospheric constant temperature drying method, following GB 5009.3-2016 “National Food Safety Standard—Determination of Moisture in Foods” [7]; the ash content was determined using the high-temperature ignition method, following GB 5009.4-2016 “National Food Safety Standard—Determination of Ash in Foods” [8]; the crude protein content was determined using the Kjeldahl method, following GB 5009.6-2016 “National Food Safety Standard—Determination of Fat in Foods” [9]; and the crude fat content was determined using the Soxhlet extraction method, referring to GB 5009.5-2016 “National Food Safety Standard—Determination of Protein in Foods” [10].

### 2.3. Determination of Amino Acid and Fatty Acid Content

Amino acids were determined following GB 5009.124-2016 “National Food Safety Standard—Determination of Amino Acids in Foods” [11]. Samples were hydrolyzed with 6 M HCl at 110 °C for 22 h under nitrogen atmosphere, then analyzed using an amino acid analyzer (S-433 Dup, SYKAM, Munich, Germany). Fatty acids were determined following GB 5009.168-2016 “National Food Safety Standard—Determination of Fatty Acids in Foods” [12]. Lipids were extracted using the Folch method (chloroform–methanol 2:1, *v*/*v*), then methylated with 14% BF3–methanol. Fatty acid methyl esters were analyzed by gas chromatography (Agilent 7890B, Santa Clara, CA, USA) equipped with a flame ionization detector and an HP-88 column (100 m × 0.25 mm × 0.20 μm). Results are expressed as g/100 g of wet muscle for amino acids, and as a percentage of total fatty acids for fatty acids.

### 2.4. Determination of Mineral Element Content

The mineral elements measured included Cu, Zn, Fe, Mn, K, Na, Ca, and Mg. Their contents were determined using inductively coupled plasma mass spectrometry (ICP-MS), following GB 5009.268-2016 “National Food Safety Standard—Determination of Multiple Elements in Foods” [13]. Samples (0.5 g) were digested with 5 mL HNO_3_ and 1 mL H_2_O_2_ in a microwave digestion system. Calibration standards were prepared from multi-element stock solutions. Detection limits ranged from 0.01 to 0.5 mg/kg. Recovery rates for certified reference material were 92–105%.

### 2.5. Nutritional Quality Evaluation

Based on the standard scoring model for amino acids per gram of nitrogen (N) recommended by the Food and Agriculture Organization/World Health Organization (FAO/WHO 2013), as well as the standard scoring model for amino acids in whole egg protein proposed by the Institute of Nutrition and Food Safety of the Chinese Academy of Preventive Medicine, chemical score (CS), the amino acid score (AAS), and essential amino acid index (EAAI) were calculated according to the following formulas:

CS = Content of a given amino acid in the protein of the test sample (mg/g)/content of the same amino acid in the protein of whole egg (mg/g);

AAS = Content of a given amino acid in the protein of the test sample (mg/g)/content of the same amino acid in the FAO/WHO standard model (mg/g);

EAAI = [(100 × t_1_/s_1_) × (100 × t_2_ /s_2_) × (100 × t_3_/s_3_) ×…× (100 × t_n_ /s_n_)]^1/n^.

In the formula: n is the number of amino acid types involved in the comparison; t_1_, t_2_, t_3_, …, t_n_ are the content of various amino acids (mg/g) in the protein of the test sample, respectively; s_1_, s_2_, s_3_, …, s_n_ are the content of the corresponding amino acids (mg/g) in the protein of whole eggs, respectively.

### 2.6. Statistical Analysis

The data are presented as mean ± standard deviation. All statistical analyses were performed using Statistical Product and Service Solutions (SPSS) 21.0. Before variance analysis, the normality of data distribution and homogeneity of variance were systematically tested to verify the prerequisites for ANOVA. For the meat yield index, a two-way ANOVA was adopted to analyze the main and interactive effects of the province and sex. For the nutritional indices, one-way ANOVA was performed based on a clear biological replicate design, with three independent biological replicates set for each province. After ANOVA, Tukey’s HSD multiple comparison test was applied for post hoc pairwise comparisons to identify significant differences among groups. A probability value of *p* < 0.05 was considered statistically significant for all tests.

## 3. Results

### 3.1. Morphological Characteristics and Meat Yield of Crayfish from Six Provinces

As shown in Table 1, the meat yield of crayfish from Jiangsu Province was significantly higher than that from the other five provinces (*p* < 0.05), while the meat yield of crayfish from Hunan and Anhui Provinces were significantly lower than those from the other provinces (*p* < 0.05). No significant differences were observed in meat yield among Henan, Hubei and Jiangxi Provinces (*p* > 0.05); however, their meat yield were significantly lower than that of Jiangsu and significantly higher than those of Anhui and Hunan (*p* < 0.05). In terms of sex, the ranking of meat yield for female crayfish was Jiangsu > Henan > Hubei > Jiangxi > Anhui > Hunan, and for males it was Jiangsu > Jiangxi > Henan > Hubei >Anhui> Hunan. Moreover, except for Anhui and Jiangxi Provinces, the meat yield of female crayfish was significantly higher than that of the males (*p* < 0.05).

### 3.2. Conventional Nutrient Components of Crayfish from Six Provinces

As shown in Table 2, crayfish from Anhui, Henan and Hubei Provinces had relatively high moisture contents of 82.3%, 82.2% and 81.2%, respectively, with no significant differences among the three (*p* > 0.05), but all were significantly higher than those from Hunan (80.6%), Jiangsu (80.2%) and Jiangxi (79.4%) (*p* < 0.05). The ash content was highest in Jiangxi (1.5%), which was not significantly different from that in Hunan (1.4%) and Jiangsu (1.4%), but these three were significantly higher than those in Henan (1.3%), Hubei (1.3%) and Anhui (1.2%) (*p* < 0.05). Regarding crude protein content, Jiangsu (18.5%), Jiangxi (18.0%), Hubei (17.0%) and Hunan (17.0%) were significantly higher than Henan (15.6%) and Anhui (16.2%) (*p* < 0.05), while no significant differences were observed among the first four provinces or between the latter two (*p* > 0.05). The crude fat content was highest in Jiangxi (0.7%), significantly higher than that in all other provinces (*p* < 0.05). Henan (0.4%), Hubei (0.3%) and Hunan (0.3%) had intermediate levels, with no significant differences among them, but all were significantly higher than those in Anhui (0.1%) and Jiangsu (0.1%) (*p* < 0.05).

### 3.3. Amino Acid Composition and Nutritional Evaluation of Crayfish Muscles from Six Provinces

#### 3.3.1. Amino Acids Composition

As shown in Table 3, 16 amino acids were detected in crayfish from all six provinces, including seven essential amino acids, two semi-essential amino acids, and seven non-essential amino acids. Among these, glutamic acid had the highest content, ranging from 2.27/100 g to 2.67 g/100 g, while methionine had the lowest content, ranging from 0.38 g/100 g to 0.44 g/100 g. Across the six provinces, Jiangsu Province exhibited the highest content for 11 amino acids; Jiangxi Province had the highest contents of methionine (Met), histidine (His), and serine (Ser); Hubei Province had the highest contents of arginine (Arg) and tyrosine (Tyr). In contrast, Henan Province had the lowest contents for 13 amino acids, while Jiangsu Province had the lowest content of histidine (His), and Anhui Province had the lowest contents of arginine (Arg) and tyrosine (Tyr). In terms of overall amino acid levels, Jiangsu Province ranked first in terms of total amino acids (TAA), total essential amino acids (EAA), total non-essential amino acids (NEAA), total flavor amino acids (DAA), and the ratio of ∑EAA/∑NEAA (%). Anhui Province ranked first in ∑EAA/∑TAA (%) and ∑DAA/∑TAA (%).

#### 3.3.2. Nutritional Evaluation

The amino acid score (AAS) and chemical score (CS) of crayfish muscle were calculated based on the FAO/WHO standard and the whole egg protein standard, respectively, and an amino acid evaluation was conducted. The results showed that among the six provinces, the AAS and CS of Lys in crayfish muscle were the highest. In Anhui Province, the AAS and CS of Leu were the highest, while the AAS of Val was the lowest across all provinces. The AAS of Ile, Leu, and Lys were all >1, and the CS of Leu and Lys in Jiangsu Province were >1. Jiangxi Province had the highest EAAI (essential amino acid index) value of 0.68, but there was no significant difference among the six provinces (*p* > 0.05). Using AAS as the evaluation criterion, Val was the first limiting amino acid in crayfish muscle for all provinces. Thr was the second limiting amino acid for crayfish muscle from Henan, Hubei, Hunan, and Jiangxi provinces, whereas Met was the second limiting amino acid for crayfish muscle from Anhui and Jiangsu provinces (Table 4).

### 3.4. Fatty Acid Composition of Crayfish from Six Provinces

As shown in Table 5, a total of 22 fatty acids were detected in the crayfish muscle from six provinces, including nine saturated fatty acids (SFAs), five monounsaturated fatty acids (MUFAs), and eight polyunsaturated fatty acids (PUFAs). Among the SFAs, C16:0 had the highest content, followed by C18:0. Among the MUFAs, C18:1n9c had the highest content, followed by C16:1; among the PUFAs, C20:5 had the highest content, followed by C20:4n6. Across different provinces, the SFA content was highest in Hubei (27.84%) and lowest in Henan (24.12%), with the other provinces ranging between 25.58% and 27.06%. The MUFA content was highest in Hunan (32.15%) and lowest in Hubei (25.04%), showing a relatively wide range of variation. The PUFA content was highest in Anhui (47.30%) and lowest in Hunan (42.19%), indicating that crayfish from Anhui had a relative advantage in PUFA accumulation. Regarding functional fatty acids, the EPA content was highest in Henan (19.6%) and lowest in Jiangxi (12.7%), showing significant regional differences. The DHA content was highest in Jiangxi (6.42%) and lowest in Henan (2.62%), while the total EPA + DHA content was highest in Hubei (22.41%) and lowest in Anhui (17.47%).

### 3.5. Mineral Element Contents of Crayfish from Six Provinces

In this study, the contents of eight mineral elements, including four macro elements and four trace elements, were measured in the muscle of crayfish from different provinces. The results showed significant regional differences. Specifically, the Cu content was highest in Jiangxi (11.2 mg/kg) and lowest in Hubei (3.38 mg/kg); the Zn content was highest in Jiangsu (16.2 mg/kg) and lowest in Hubei (13.4 mg/kg); the Fe content exhibited considerable variation, with the highest value in Hunan (9.06 mg/kg) and the lowest in Hubei (2.28 mg/kg); the Mn content was highest in Henan (3.72 mg/kg) and lowest in Hunan (1.77 mg/kg); the K content was highest in Jiangsu (4.24 × 10^3^ mg/kg) and lowest in Anhui (3.14 × 10^3^ mg/kg); the Ca content was highest in Jiangxi (1.28 × 10^3^ mg/kg) and lowest in Jiangsu (342 mg/kg); the Mg content was highest in Jiangsu (364 mg/kg) and lowest in Henan (268 mg/kg) (Table 6).

## 4. Discussion

### 4.1. Comparison of Meat Yield and Conventional Nutrient Contents in Crayfish from Different Provinces

Meat yield is a core indicator for assessing the edible portion ratio and production performance of crayfish that is comprehensively regulated by multiple factors, including stocking density, dietary nutrient levels, and water environment conditions. In this study, significant regional differences in crayfish meat yield were observed among the six sampling provinces. Specifically, Jiangsu crayfish exhibited the highest meat content, with values of 22.97% ± 3.10% in females and 20.72% ± 5.13% in males. By comparison, crayfish from Hunan and Anhui showed relatively lower meat content. In Hunan Province, the meat yield of female and male crayfish was 15.40% ± 1.06% and 12.92% ± 1.36%, respectively, while that in Anhui Province was 15.84% ± 3.39% for females and 13.93% ± 4.73% for males. Such spatial variations in meat yield may be largely attributed to regional farming and environmental differences. Located in the middle-lower Yangtze River region, Jiangsu possesses a dense river network with efficient water exchange and sufficient dissolved oxygen in paddy fields, providing favorable habitat conditions for muscle growth and development [14]. Furthermore, the locally optimized stocking density alleviates intraspecific competition, and matched with high-quality feed management, facilitates effective muscle nutrient accumulation and tissue development [15]. In contrast, the locally optimized stocking density alleviates intraspecific competition, and matched with high-quality feed management, facilitates effective muscle nutrient accumulation and tissue development.

From the perspective of sexual differences, the female crayfish generally exhibited higher meat contents than males across the six surveyed provinces. Extremely significant differences in meat yield between females and males were observed in Hubei and Hunan Provinces, while significant sexual differences were also detected in Jiangsu Province. This phenomenon conformed to the physiological characteristics of the crayfish. After sexual maturation, female crayfish prioritized muscle tissue development and nutrient accumulation. In contrast, male individuals allocated more energy to cheliped growth and locomotor activity, which restricts somatic muscle deposition and consequently results in a lower meat proportion [16]. Additionally, no significant differences in meat yield were found among Henan, Hubei, and Jiangxi Provinces, possibly due to their geographical proximity, similar climatic conditions, and high homogeneity in farming practices, which led to minor variations in growth environment and nutrient supply levels, ultimately reflecting insignificant spatial variation in meat content.

Regional variations in conventional nutritional components of crayfish muscle further reflected the influence of environmental conditions and farming management [17]. In the present study, Jiangxi Province exhibited the highest crude fat content (0.7%), significantly higher than other provinces. This phenomenon may be attributed to a higher proportion of oil added to commercial feeds or the abundant lipid-rich natural prey (e.g., zooplankton and aquatic insects) in rice paddies, resulting in increased fat accumulation during energy metabolism. In comparison, the crude fat contents of crayfish in Jiangsu and Anhui Provinces were only 0.1%, which was likely due to the application of high-protein-oriented feeding regimes and the relatively spacious aquatic environment, which increases locomotor energy consumption, shifts the dominant metabolic pattern toward protein utilization, and consequently reduces lipid accumulation in muscle tissue [18].

In terms of crude protein content, Jiangsu Province presented the highest value of 18.5% ± 0.35%, whereas Henan Province showed the lowest content at 15.6% ± 0.21%. The spatial variation in protein accumulation may be attributed to the differences in protein source quality and dietary formula composition. In Jiangsu, aquaculture feeds often use high-quality protein ingredients such as fish meal and soybean meal, whose balanced amino acid profiles closely matched the physiological growth requirements of crayfish, thereby effectively facilitating muscle protein synthesis and deposition. In contrast, the feeds applied in Henan contained a high proportion of plant-derived protein, which exhibited low digestibility and absorption efficiency in crayfish, ultimately limiting muscle protein accumulation [19]. Additionally, Henan recorded the highest muscle moisture content (82.2%), while Jiangxi recorded the lowest (79.4%). Such differences were closely associated with muscle tissue structure characteristics: crayfish with higher moisture content tend to have coarser muscle fibers and larger intercellular spaces, whereas those with lower moisture content possess denser and firmer muscle texture, which may further affect the taste quality of crayfish [20]. Moreover, Jiangxi exhibited the highest ash content (1.5%), which was consistent with the relatively higher contents of Cu, Ca, Na and other mineral elements in the mineral component analysis of Jiangxi samples (see later), indicating that the regional disparity in ash content was mainly determined by the differential mineral accumulation capacity of crayfish from different producing areas.

### 4.2. Amino Acid Composition and Nutritional Value Evaluation of Crayfish Muscle from Different Provinces

In this study, a total of 16 amino acids were detected in all samples, among which glutamic acid being the highest and methionine the lowest, which is consistent with the general amino acid distribution characteristics of most aquatic products. As a key flavor-enhancing amino acid, the high accumulation of glutamic acid was the core factor contributing to the delicious flavor of crayfish [21,22]. In terms of amino acid nutritional quality, crayfish from Jiangsu Province presented prominent superiority, with the highest contents of 11 amino acids across all provinces]. Among them, the contents of total amino acids (16.59 ± 3.08 g/100 g), total essential amino acids (6.18 ± 1.45 g/100 g), and total flavor amino acids (6.17 ± 1.78 g/100 g) were all the highest. Meanwhile, the ∑EAA/∑NEAA% reached 77.74 ± 6.94%, which conformed to the ideal ratio range (0.6–1.0) recommended by FAO/WHO. This indicated that the crayfish from Jiangsu Province possessed superior protein quality, with both high nutritional value and excellent flavor characteristics. This prominent advantage may be attributed to the abundance of natural bait (e.g., algae, small invertebrates) in the local aquaculture environment, which provided sufficient and balanced amino acid precursors for crayfish. In addition, the scientifically formulated amino acid composition of the feeds further satisfied the physiological requirements for essential amino acid synthesis. In contrast, crayfish from Henan Province performed the poorest regarding the contents of 13 amino acids among the six provinces, with a total amino acid content of only 13.94 ± 2.34 g/100 g. This phenomenon may be related to local water quality parameters (e.g., pH, hardness) or to incomplete amino acid profiles or unbalanced amino acid profiles in the feeds, which hindered the synthesis and accumulation in crayfish muscle [23]. Furthermore, obvious regional specificity in individual amino acid contents was observed: Jiangxi Province showed the highest contents of methionine, histidine, and serine, while Hubei Province exhibited the maximum levels of arginine and tyrosine. These regional advantages in specific amino acids may be associated with variations in trace element contents in the soil and water. Some trace elements served as co-enzymes for amino acid synthesis enzymes, and their regional differences can directly regulate the synthesis efficiency of specific amino acids [24].

The amino acid evaluation results revealed valine (Val) as the primary limiting amino acid for crayfish in all six provinces, presenting the lowest AAS values of (0.62–0.67). This finding matched the typical limiting amino acid pattern observed in most freshwater shrimp species. Threonine (Thr) served as the secondary limiting amino acid for crayfish sampled from Henan, Hubei, Hunan and Jiangxi Provinces, with AAS values between 0.73 and 0.75. By contrast, methionine (Met) was the second limiting amino acid in Anhui and Jiangsu Provinces, with AAS values of 0.72–0.74. These results implied that Val, Thr or Met tailored to regional conditions can further elevate the nutritional quality of muscle protein [25]. The essential amino acid index (EAAI) values among different provinces fluctuated narrowly from 0.66 to 0.68 without significant differences, yet these absolute values remained comparatively low. This indicated that crayfish muscle protein quality was inferior to whole egg protein, leaving considerable potential for nutritional improvement. Further progress can be achieved via refined optimization of feed nutrient formulations in subsequent farming practices [26].

### 4.3. Comparison of Fatty Acid Contents in Crayfish Muscle from Different Provinces

Crayfish muscle possesses abundant fatty acid profiles dominated by unsaturated fatty acids, endowing it with favorable nutritional properties [27]. In this study, crayfish from Anhui Province contained the highest PUFA content (47.30%), markedly exceeding values from other provinces, especially with C20:4n6 content reaching 13.2 ± 1.15%. This indicated that the aquaculture environment in Anhui was more conducive for PUFA deposition. It was inferred that the local paddy fields were abundant in phytoplankton (e.g., diatoms and green algae), which acted as critical natural sources of PUFAs. By ingesting these algae, crayfish could acquire sufficient precursors to synthesize polyunsaturated fatty acids endogenously [28]. Furthermore, parts of Anhui may adopt a model of crayfish and submerged macrophytes, which are rich in unsaturated fatty acids, supplying additional nutritional substrates for crayfish lipid accumulation [29]. Hunan Province exhibited the highest MUFA content (32.15%), where C18:1n9c accounted for 25.6 ± 2.68%. This may stem from the relatively higher water temperature in Hunan’s culture zones. Elevated water temperature boosted the activity of fatty acid synthases, particularly those responsible for MUFA biosynthesis. Additionally, feeds supplemented with large proportions of plant oils (rapeseed oil, peanut oil, etc.) also facilitate greater MUFA deposition in crayfish muscle [30].

Significant regional disparities were observed in the functional fatty acid profiles of crayfish muscle. Henan Province exhibited the highest EPA content (19.6 ± 1.54%), which was 1.54-fold that of Jiangxi Province (12.7 ± 1.13%). In contrast, Jiangxi Province had the highest DHA content (6.42 ± 0.57%), reaching 2.45-fold that of Henan Province (2.62 ± 0.35%). Such distinct spatial variations may be attributed to inter-population genetic differences in fatty acid synthesis capacity. Crayfish populations from different provinces exhibited variable activities of key enzymes involved in long-chain polyunsaturated fatty acid biosynthesis, such as Δ6-desaturase. Meanwhile, regional differences in the environmental availability of EPA and DHA precursors (e.g., C18:3n-3) further modulated the final accumulation of functional fatty acids in muscle tissue [31]. Notably, the PUFA/SFA ratios of crayfish from all six provinces were consistently higher than 1, satisfying the optimal fatty acid ratio criteria for human healthy diets recommended by nutritional guidelines. In particular, Hubei and Anhui Provinces presented relatively higher PUFA/SFA values of 1.69 and 1.84, respectively. These findings demonstrated that rice field-cultured crayfish possessed superior fatty acid nutritional quality.

### 4.4. Comparison of Mineral Contents in Crayfish Muscle from Different Provinces

Mineral accumulation in aquatic organisms is highly dependent on ambient farming conditions, particularly the elemental background values of field water and soil substrates [32]. In this study, eight mineral elements (Cu, Zn, Fe, Mn, K, Na, Ca, Mg) detected in crayfish muscle exhibited significant regional differences, which were regulated by local water quality, soil geochemistry, and dietary nutrient composition [33]. As exogenous nutrients, minerals cannot be autonomously synthesized by crayfish and were primarily accumulated via water osmotic absorption and food ingestion [34].

Among all sampling regions, Jiangxi Province showed prominent mineral accumulation capacity, with the highest contents of Cu, Na, and Ca across the six provinces, with Ca content being 3.74 times that of Jiangsu Province. This superior mineral enrichment was closely associated with the unique soil and hydrological characteristics of Jiangxi. The region is mainly covered by red soil, which possesses high background values of Ca, Cu, and other elements [35]. Furthermore, the paddy field water presents relatively high hardness with abundant Ca. Crayfish can efficiently absorb large amounts of Ca through gill respiration and body surface osmosis, while the ingestion of benthic organisms further facilitates mineral deposition in muscle tissue. Hunan Province had the highest Fe content, whereas Hubei Province recorded the lowest value, with a 3.97-fold difference between the two regions. This may be attributed to the differential iron bioavailability in ambient water environments. Some aquaculture areas in Hunan are adjacent to river and lake water systems, where elevated organic matter content enables iron ions to form easily absorbable complexes for crayfish uptake. In contrast, the relatively higher pH of paddy water in parts of Hubei promotes iron ion precipitation, thereby reducing the bioavailability of iron and limiting its accumulation in crayfish muscle [36].

Jiangsu Province exhibited distinct advantages in K and Mg contents. It was presumably attributed to the relatively high concentrations of potassium and magnesium ions in local paddy water, which mainly result from the routine application of potassium and magnesium fertilizers in farmland soil [37]. Meanwhile, the addition of mineral premix in feed further provided a stable and continuous supply of K and Mg for crayfish growth. These macroelements are essential for maintaining the physiological metabolism and homeostasis in crayfish and also serve as an excellent dietary electrolyte source for the human body [38]. In addition, crayfish from Henan Province possessed the highest Mn content, significantly higher than that of Hunan Province. This difference may be related to the geochemical characteristics of local soil. Cinnamon soil, widely distributed in parts of Henan, is inherently rich in Mn. Mn can migrate from soil to water and feed, and are eventually accumulated in crayfish muscle, leading to higher Mn deposition in Henan crayfish.

Overall, the crayfish muscle collected from the six surveyed provinces was rich in essential mineral elements, with Zn, Fe, Ca contents ranging from 13.4 to 16.2 mg/kg, 2.28 to 9.06 mg/kg, and 342 to 1280 mg/kg, respectively. These minerals participate in diverse vital physiological processes in the human body. Specifically, Zn modulates the activity of immune-related enzymes, Fe is essential for hemoglobin synthesis, and Ca plays a crucial role in bone growth and development. These characteristics confirm that paddy-field-cultured crayfish constitute a high-quality dietary source for human mineral supplementation [39]. Furthermore, all detected mineral contents were within the permissible limits specified by national food safety standards, verifying the excellent food safety of rice–crayfish coculture products (Table 7).

## 5. Conclusions

This study systematically compared and analyzed the meat yield, conventional nutrient components, amino acid composition, fatty acid composition, and mineral element contents of crayfish collected from six major producing provinces in China, revealing significant regional variations in the nutritional quality of crayfish from different geographical origins, with distinct phenotypic advantages in each producing area. Jiangsu crayfish Province presented superior performance in meat rate, crude protein content, and amino acid content. Jiangxi samples exhibited outstanding crude fat and Ca contents, while Anhui crayfish showed prominent advantage in PUFA accumulation. Hubei Province recorded the highest total EPA and DHA contents, Henan crayfish possessed the highest manganese and EPA levels, and Hunan samples were characterized by the maximum iron content. Despite the regional differences in individual nutritional indicators, all six geographical populations exhibited consistent and superior basic nutritional characteristics. All tested crayfish were high in protein and low in fat, with balanced amino acid compositions, reasonable fatty acid structures, and abundant essential mineral elements. Moreover, all detected mineral element concentrations were within the permissible limits specified by national food safety standards, indicating that the crayfish were safe for consumption with respect to these elements across the six provinces. The limitations of this study lied in the fact that the sample collection only covers six major aquaculture provinces and did not directly measure aquaculture environmental factors (such as water pH, hardness, soil mineral content) and feed nutrient composition; thus, it was not possible to establish a quantitative relationship between these factors and the nutritional differences in crayfish. Future research could expand the coverage of production areas, increase the number of samples and the collection seasons, simultaneously measure aquaculture environmental factors and feed composition, conduct correlation analysis, and identify the dominant factors contributing to regional differences in nutritional composition.

## Figures and Tables

**Table 1:** The weight and meat yield of crayfish samples from six provinces.

Area	Body Weight (g)		Meat Yield (%)	
	♀	♂	♀	♂
Henan	30.75 ± 4.99	33.28 ± 5.25	19.99 ± 2.32 ^Ab^	17.42 ± 3.11 ^Bb^
Hubei	24.00 ± 2.67	25.62 ± 3.63	19.77 ± 2.66 ^Ab^	16.55 ± 3.05 ^Bb^
Hunan	31.86 ± 2.39	32.73 ± 3.89	15.40 ± 1.06 ^Ac^	12.92 ± 1.36 ^Bc^
Anhui	27.70 ± 7.79	23.16 ± 7.53	15.84 ± 3.39 ^Ac^	13.93 ± 4.73 ^Ac^
Jiangsu	19.29 ± 6.07	19.10 ± 7.07	22.97 ± 3.10 ^Aa^	20.72 ± 5.13 ^Ba^
Jiangxi	18.33 ± 7.09	15.74 ± 2.05	18.77 ± 2.16 ^Ab^	17.80 ± 2.00 ^Ab^

Note: Different uppercase superscript letters (A, B) indicate significant differences between sexes within the same row (*p* < 0.05). Different lowercase superscript letters (a, b, c) indicate significant differences among provinces within the same column (*p* < 0.05). **♀** and **♂** denoted female and male, respectively.

**Table 2:** Conventional nutrient contents of crayfish samples from different provinces (g/100 g).

Area	Moisture	Ash	Crude protein	Crude Fat
Henan	82.2 ± 1.15 ^a^	1.3 ± 0.03 ^b^	15.6 ± 0.11 ^b^	0.4 ± 0.02 ^b^
Hubei	81.2 ± 1.07 ^a^	1.3 ± 0.04 ^b^	17.0 ± 0.34 ^a^	0.3 ± 0.01 ^b^
Hunan	80.6 ± 1.10 ^b^	1.4 ± 0.05 ^a^	17.0 ± 0.29 ^a^	0.3 ± 0.01 ^b^
Anhui	82.3 ± 1.21 ^a^	1.2 ± 0.04 ^b^	16.2 ± 0.25 ^b^	0.1 ± 0.01 ^c^
Jiangsu	80.2 ± 1.09 ^b^	1.4 ± 0.05 ^a^	18.5 ± 0.43 ^a^	0.1 ± 0.01 ^c^
Jiangxi	79.4 ± 0.09 ^b^	1.5 ± 0.06 ^a^	18.0 ± 0.31 ^a^	0.7 ± 0.03 ^a^

Note: Different lowercase superscript letters indicate significant differences (*p* < 0.05) within the same column.

**Table 3:** Amino acid composition and contents of crayfish samples from different provinces (wet weight).

Amino Acids		Henan	Hubei	Hunan	Anhui	Jiangsu	Jiangxi
EAA (g/100 g)	Thr	0.58 ± 0.02	0.64 ± 0.02	0.64 ± 0.02	0.63 ± 0.04	0.66 ± 0.02	0.65 ± 0.02
	Val	0.62 ± 0.02	0.68 ± 0.02	0.68 ± 0.02	0.68 ± 0.02	0.71 ± 0.02	0.70 ± 0.02
	Met	0.38 ± 0.01	0.42 ± 0.02	0.42 ± 0.01	0.38 ± 0.01	0.42 ± 0.02	0.44 ± 0.01
	Ile	0.62 ± 0.02	0.70 ± 0.01	0.71 ± 0.02	0.70 ± 0.02	0.76 ± 0.02	0.72 ± 0.02
	Leu	1.14 ± 0.02	1.28 ± 0.02	1.30 ± 0.44	1.24 ± 0.23	1.38 ± 0.03	1.32 ± 0.03
	Phe	0.66 ± 0.01	0.72 ± 0.03	0.74 ± 0.03	0.69 ± 0.02	0.77 ± 0.02	0.78 ± 0.02
	Lys	1.23 ± 0.06	1.38 ± 0.06	1.38 ± 0.05	1.24 ± 0.05	1.48 ± 0.08	1.44 ± 0.06
SEAA (g/100 g)	His	0.42 ± 0.03	0.39 ± 0.03	0.40 ± 0.03	0.37 ± 0.03	0.37 ± 0.03	0.44 ± 0.04
	Arg	1.46 ± 0.17 ^b^	1.74 ± 0.34 ^a^	1.68 ± 0.23 ^a^	1.22 ± 0.15 ^c^	1.48 ± 0.13 ^b^	1.64 ± 0.25 ^a^
NEAA (g/100 g)	Asp *	1.40 ± 0.12 ^b^	1.60 ± 0.18 ^a^	1.62 ± 0.21 ^a^	1.58 ± 0.22 ^b^	1.70 ± 0.25 ^a^	1.66 ± 0.26 ^a^
	Ser	0.54 ± 0.01 ^a^	0.61 ± 0.02 ^a^	0.61 ± 0.03 ^a^	0.54 ± 0.02 ^a^	0.58 ± 0.02 ^a^	0.62 ± 0.03 ^a^
	Glu *	2.27 ± 0.21	2.64 ± 0.32	2.59 ± 0.22	2.51 ± 0.24	2.67 ± 0.33	2.60 ± 0.36
	Gly *	0.66 ± 0.02	0.72 ± 0.03	0.76 ± 0.04	0.78 ± 0.05	0.78 ± 0.05	0.76 ± 0.04
	Ala *	0.92 ± 0.05 ^a^	0.96 ± 0.05 ^a^	0.97 ± 0.05 ^a^	0.96 ± 0.05 ^a^	1.02 ± 0.04 ^a^	1.00 ± 0.05 ^a^
	Tyr	0.60 ± 0.02 ^b^	0.70 ± 0.03 ^a^	0.68 ± 0.02 ^a^	0.54 ± 0.01 ^b^	0.66 ± 0.02 ^a^	0.68 ± 0.02 ^a^
	Pro	0.46 ± 0.01 ^a^	0.48 ± 0.01 ^a^	0.50 ± 0.01 ^a^	0.50 ± 0.01 ^a^	0.54 ± 0.01 ^a^	0.49 ± 0.01 ^a^
∑EAA (g/100 g)		5.23 ± 0.95 ^c^	5.82 ± 0.78 ^b^	5.87 ± 1.23 ^b^	5.56 ± 1.01 ^b^	6.18 ± 1.45 ^a^	6.05 ± 1.33 ^a^
∑DAA (g/100 g)		5.25 ± 1.02 ^b^	5.92 ± 1.01 ^a^	5.94 ± 0.99 ^a^	5.83 ± 1.21 ^a^	6.17 ± 1.78 ^a^	6.02 ± 1.56 ^a^
∑NEAA (g/100 g)		6.85 ± 1.07 ^b^	7.71 ± 1.22 ^a^	7.73 ± 1.56 ^a^	7.41 ± 1.64 ^a^	7.95 ± 1.89 ^a^	7.81 ± 1.96 ^a^
∑TAA (g/100 g)		13.94 ± 2.34 ^b^	15.66 ± 2.75 ^a^	15.68 ± 2.74 ^a^	14.59 ± 2.51 ^b^	16.59 ± 3.08 ^a^	15.94 ± 2.71 ^a^
∑EAA/∑NEAA%		76.35 ± 4.65	75.49 ± 5.13	75.94 ± 6.07	75.03 ± 5.28	77.74 ± 6.94	77.46 ± 6.58
∑DAA/∑TAA%		37.52 ± 2.55 ^a^	37.16 ± 2.67 ^a^	37.44 ± 2.73 ^a^	38.11 ± 3.01 ^a^	37.25 ± 2.56 ^a^	37.95 ± 3.24 ^a^

Note: * indicates flavor amino acids. Different lowercase superscript letters indicate significant differences (*p* < 0.05) within the same row.

**Table 4:** Evaluation of essential amino acid composition of crayfish samples from different provinces.

EAA	FAO/ WHO Pattern	Whole Hen’s Egg Pattern	Henan		Hubei		Hunan		Anhui		Jiangsu		Jiangxi	
			AAS	CS	AAS	CS	AAS	CS	AAS	CS	AAS	CS	AAS	CS
Ile	40	54	1.11	0.82	1.12	0.83	1.13	0.84	1.20	0.89	1.15	0.85	1.13	0.84
Leu	70	86	1.17	0.95	1.17	0.95	1.19	0.97	1.22	0.99	1.25	1.01	1.19	0.97
Thr	40	47	0.75	0.64	0.73	0.63	0.73	0.62	0.77	0.66	0.73	0.62	0.73	0.63
Phe+ Tyr	60	93	0.79	0.51	0.82	0.53	0.81	0.52	0.76	0.49	0.79	0.51	0.82	0.53
Lys	55	70	1.25	0.99	1.26	0.99	1.26	0.99	1.21	0.95	1.28	1.01	1.30	1.02
Val	50	66	0.62	0.47	0.62	0.47	0.62	0.47	0.67	0.51	0.63	0.48	0.63	0.48
Met	35	57	0.78	0.48	0.77	0.47	0.77	0.47	0.74	0.46	0.72	0.44	0.79	0.48
EAAI			0.66		0.67		0.67		0.67		0.67		0.68	

**Table 5:** Fatty acid composition of crayfish samples from six provinces.

Fatty Acids	Henan	Hubei	Hunan	Anhui	Jiangsu	Jiangxi
C14:0	0.645 ± 0.05 ^a^	0.593 ± 0.05 ^a^	0.453 ± 0.03 ^b^	0.338 ± 0.02 ^c^	0.454 ± 0.04 ^b^	0.469 ± 0.04 ^b^
C16:0	10.6 ± 1.02 ^c^	11.9 ± 1.23 ^b^	12.0 ± 1.24 ^a^	10.9 ± 0.85 ^c^	11.8 ± 1.03 ^b^	11.4 ± 1.05 ^b^
C16:1	6.00 ± 0.46 ^a^	4.12 ± 0.32 ^b^	3.14 ± 0.25 ^c^	3.62 ± 0.29 ^c^	4.34 ± 0.39 ^b^	2.66 ± 0.19 ^d^
C17:0	0.708 ± 0.06 ^c^	1.86 ± 0.01 ^a^	1.10 ± 0.01 ^b^	1.64 ± 0.02 ^a^	1.35 ± 0.01 ^b^	1.12 ± 0.01 ^b^
C17:1	0.655 ± 0.04 ^c^	0.902 ± 0.07 ^a^	0.613 ± 0.05 ^c^	0.781 ± 0.06 ^b^	0.940 ± 0.08 ^a^	0.610 ± 0.05 ^c^
C18:0	5.80 ± 0.45 ^b^	7.32 ± 0.58 ^a^	7.00 ± 0.51 ^a^	7.06 ± 0.49 ^a^	7.92 ± 0.61 ^a^	7.24 ± 0.56 ^a^
C18:1n9c	22.8 ± 2.53 ^b^	17.6 ± 1.46 ^d^	25.6 ± 2.68 ^a^	21.0 ± 2.24 ^b^	21.1 ± 1.96 ^b^	21.1 ± 2.39 ^b^
C18:2n6c	7.24 ± 0.56 ^c^	8.87 ± 0.67 ^c^	9.94 ± 0.82 ^b^	10.8 ± 1.1 ^b^	11.5 ± 1.45 ^b^	13.8 ± 2.32 ^a^
C18:3n3	3.79 ± 0.28 ^b^	5.27 ± 0.43 ^a^	3.01 ± 0.24 ^b^	3.44 ± 0.29 ^b^	5.54 ± 0.46 ^a^	2.75 ± 0.18 ^c^
C20:0	0.808 ± 0.06 ^b^	1.00 ± 0.09 ^b^	0.906 ± 0.06 ^b^	1.14 ± 0.04 ^a^	1.26 ± 0.09 ^a^	1.04 ± 0.05 ^a^
C20:1	1.40 ± 0.08 ^b^	1.10 ± 0.07 ^b^	2.04 ± 0.12 ^a^	0.297 ± 0.01 ^d^	0.888 ± 0.05 ^c^	1.82 ± 0.09 ^a^
C20:2	1.04 ± 0.11 ^c^	1.06 ± 0.09 ^c^	1.22 ± 0.11 ^b^	1.26 ± 0.12 ^b^	1.07 ± 0.01 ^c^	1.33 ± 0.02 ^a^
C20:4n6	9.16 ± 0.83 ^b^	8.51 ± 0.74 ^c^	7.12 ± 0.66 ^c^	13.2 ± 1.15 ^a^	7.37 ± 0.69 ^c^	8.50 ± 0.72 ^c^
C22:0	1.42 ± 0.17 ^a^	1.40 ± 0.11 ^a^	1.18 ± 0.16 ^b^	1.08 ± 0.12 ^b^	0.854 ± 0.06 ^c^	1.53 ± 0.13 ^a^
C13:0	2.92 ± 0.13 ^a^	1.97 ± 0.11 ^c^	1.72 ± 0.13 ^c^	2.14 ± 0.19 ^b^	2.34 ± 0.15 ^b^	1.80 ± 0.09 ^c^
C15:0	0.503 ± 0.04 ^c^	0.954 ± 0.06 ^a^	0.668 ± 0.05 ^b^	0.654 ± 0.05 ^b^	0.548 ± 0.03 ^c^	0.922 ± 0.08 ^a^
C15:1	0.352 ± 0.02 ^c^	1.32 ± 0.89 ^a^	0.754 ± 0.06 ^b^	1.33 ± 0.01 ^a^	0.834 ± 0.06 ^b^	0.731 ± 0.06 ^b^
C18:3	0.374 ± 0.02 ^c^	0.473 ± 0.02 ^c^	0.336 ± 0.02 ^c^	0.746 ± 0.05 ^a^	0.850 ± 0.06 ^a^	0.580 ± 0.04 ^b^
C21:0	0.716 ± 0.05 ^b^	0.836 ± 0.06 ^a^	0.556 ± 0.03 ^c^	0.728 ± 0.05 ^b^	0.519 ± 0.02 ^c^	0.922 ± 0.07 ^a^
C20:3	1.62 ± 0.01 ^a^	0.422 ± 0.02 ^c^	0.482 ± 0.02 ^c^	0.384 ± 0.01 ^c^	0.604 ± 0.03 ^b^	0.657 ± 0.03 ^b^
C20:5 (EPA)	19.6 ± 1.54 ^a^	16.8 ± 1.62 ^b^	16.0 ± 1.59 ^b^	13.4 ± 1.36 ^c^	13.4 ± 1.28 ^c^	12.7 ± 1.13 ^c^
C22:6 (DHA)	2.62 ± 0.35 ^c^	5.61 ± 0.46 ^a^	4.08 ± 0.39 ^b^	4.07 ± 0.41 ^b^	4.42 ± 0.51 ^b^	6.42 ± 0.57 ^a^
SFA	24.12 ^b^	27.84 ^a^	25.58 ^b^	25.69 ^b^	27.06 ^a^	26.45 ^a^
MUFA	31.21 ^a^	25.04 ^c^	32.15 ^a^	27.03 ^b^	28.1 ^b^	26.92 ^b^
PUFA	45.42 ^b^	47.02 ^a^	42.19 ^c^	47.3 ^a^	44.75 ^b^	46.74 ^a^
EPA + DHA	22.22 ^a^	22.41 ^a^	20.08 ^b^	17.47 ^c^	17.82 ^c^	19.12 ^b^

Note: Different lowercase superscript letters indicate significant differences (*p* < 0.05) within the same row.

**Table 6:** Major mineral contents of crayfish samples from six provinces (mg/kg).

Elements	Henan	Hubei	Hunan	Anhui	Jiangsu	Jiangxi
Cu	5.14 ± 1.56 ^c^	3.38 ± 1.32 ^c^	9.28 ± 2.68 ^b^	8.50 ± 2.54 ^b^	3.74 ± 1.69 ^c^	11.2 ± 3.11 ^a^
Zn	14.0 ± 1.52 ^b^	13.4 ± 1.31 ^b^	14.8 ± 1.65 ^b^	16.0 ± 1.97 ^a^	16.2 ± 2.15 ^a^	15.4 ± 1.91 ^a^
Fe	3.79 ± 0.93 ^d^	2.28 ± 0.65 ^d^	9.06 ± 1.77 ^a^	8.32 ± 1.56 ^a^	7.50 ± 1.54 ^b^	5.94 ± 1.22 ^c^
Mn	3.72 ± 1.11 ^a^	2.51 ± 0.72 ^b^	1.77 ± 0.27 ^b^	3.62 ± 0.93 ^a^	2.00 ± 0.85 ^b^	3.64 ± 1.23 ^a^
K	3260 ± 123 ^c^	3670 ± 141 ^b^	3440 ± 129 ^c^	3140 ± 105 ^c^	4240 ± 178 ^a^	3700 ± 154 ^b^
Na	1230 ± 55 ^b^	966 ± 41 ^c^	1280 ± 69 ^b^	1240 ± 65 ^b^	726 ± 32 ^c^	1420 ± 83 ^a^
Ca	655 ± 29 ^b^	412 ± 18 ^c^	604 ± 31 ^b^	590 ± 25 ^b^	342 ± 11 ^c^	1280 ± 76 ^a^
Mg	268 ± 18 ^c^	276 ± 18 ^c^	278 ± 21 ^c^	280 ± 26 ^c^	364 ± 31 ^a^	300 ± 29 ^b^

Note: Different lowercase superscript letters indicate significant differences (*p* < 0.05) within the same line.

**Table 7:** Summary of advantages of crayfish from six major producing provinces in China.

Province	Advantages
Henan	Outstanding accumulation of manganese (Mn) and EPA
Hubei	Optimal comprehensive performance of functional fatty acids (EPA + DHA)
Hunan	Significant advantages in iron (Fe) and monounsaturated fatty acid (MUFA)
Anhui	Significant advantages in polyunsaturated fatty acid (PUFA) nutrition
Jiangsu	Superior meat yield, protein nutrition, and comprehensive amino acid quality
Jiangxi	Strongest fat nutrition and mineral enrichment capacity

## Data Availability

The original contributions presented in this study are included in the article. Further inquiries can be directed to the corresponding author.
